# Embryogenesis in *Polianthes tuberosa* L var. Simple: from megasporogenesis to early embryo development

**DOI:** 10.1186/s40064-016-3528-z

**Published:** 2016-10-18

**Authors:** Alejandra G. González-Gutiérrez, Benjamín Rodríguez-Garay

**Affiliations:** Unidad de Biotecnología Vegetal, Centro de Investigación y Asistencia en Tecnología y Diseño del Estado de Jalisco, A.C. Camino Arenero 1227, El Bajío del Arenal, 45019 Zapopan, Jalisco Mexico

**Keywords:** Monosporic, Polygonum-type, Central cell, Helobial endosperm, Postament, Hypostase, Zygotic embryo, Confocal microscopy, Feulgen staining

## Abstract

The genus *Polianthes* belongs to the subfamily Agavoideae of the Asparagaceae family formerly known as Agavaceae. The genus is endemic to México and comprises about 15 species, among them is *Polianthes tuberosa* L. The aim of this work was to study and characterize the embryo sac and early embryo development of this species in order to generate basic knowledge for its use in taxonomy, in vitro fertilization and production of haploid plants and to complement studies already performed in other genera and species belonging to the Agavoideae sub-family. It was found that the normal development of the *P. tuberosa* var. Simple embryo sac follows a monosporic pattern of the Polygonum type and starts its development from the chalazal megaspore. At maturity, the embryo sac is of a pyriform shape with a chalazal haustorial tube where the antipodals are located, just below the hypostase, which connects the embryo sac with the nucellar tissue of the ovule. The central cell nucleus shows a high polarity, being located at the chalazal extreme of the embryo sac. The position of cells inside the *P. tuberosa* embryo sac may be useful for in depth studies about the double fertilization. Furthermore, it was possible to make a chronological description of the events that happen from fertilization and early embryo development to the initial development of the endosperm which was classified as of the helobial type.

## Background

The genus *Polianthes* belongs to the subfamily Agavoideae of the Asparagaceae family formerly known as Agavaceae (APG III 2009). The genus is endemic to México and comprises about 15 species, among them *Polianthes tuberosa* L. (Solano and Feria 2007; García-Mendoza and Galván 1995). It is an important economical plant because of its use as an ornamental plant and because its essential oils are highly appreciated for the manufacture of perfumes and other essences (Benschop 1993; Sangavai and Chellapandi 2008; Hodges 2010; Barba-Gonzalez et al. 2012). The species is commercially propagated by asexual methods which have reduced its genetic variability, thus reducing flower forms, sizes and colors (Shillo 1992), as well as increasing its vulnerability to biotic and abiotic stress (Hernández-Mendoza et al. 2015).

Embryological studies comprising the formation of male and female gametes, double fertilization and embryo development and endosperm (Maheshwari 1950) allow the understanding of factors that control the processes of embryonic development in order to manipulate them for practical applications (Bhojwani and Bhatnagar 1983).

In this regard, the female gametophyte plays a critical role in every stage of the reproductive process such as the direction of pollen tube growth towards the egg (Higashiyama 2002), the transport of sperm nuclei of the embryo sac through the central and egg cells in the process of double fertilization (Lord and Russell 2002; Russell 1993; Huang and Russell 1992; Ye et al. 2002; Weterings and Russell 2004), and once the fertilization is completed, genes expressed in the maternal tissue are involved in embryo and endosperm development (Ray 1997; Chaudhury and Berger 2001).

Most of the studies on the embryogenesis of Asparagaceae describe the embryo sac development as of the Monosporic–Polygonum type such as in *Yucca rupicola* (Watkins 1937), *Yucca aloifolia* (Wolf 1940), *Agave lechuguilla* (Grove 1941), *Agave virginica* (Regen 1941), *Hesperocallis undulata* and *Leucocrinum montanum* (Cave 1948), *Comospermum yedoense* (Rudall 1999), *Agave tequilana* (Escobar-Guzmán et al. 2008; González-Gutiérrez et al. 2014), *Yucca elephantipes* (Cruz-Cruz 2013) and *Yucca filamentosa* (Reed 1903), being the exception *Agave fourcroydes* and *Agave angustifolia* (Piven et al. 2001) in which the embryo sac was reported as bisporic of the *Allium* type. Knowledge about embryo and endosperm development in the sub-family Agavaoideae is limited. In 1941 Regen described the endosperm of *A. virginica* as of the nuclear type, while Gonzalez-Gutiérrez et al. (2014) reported the endosperm of *A. tequilana* as of the helobial type. However, reports about female gametophyte development, fertilization and embryo development in the genus *Polianthes* specifically in the species *P. tuberosa* are not available.

The aim of this work was to study and characterize such processes in order to generate basic knowledge for its use in taxonomy, in vitro fertilization and production of haploid plants among other uses. Furthermore, to complement studies already performed in other genera and species belonging to the Agavoideae sub-family.

## Methods

### Plant material

The plant material that was used in this work consisted of bulbs of *P. tuberosa* var. Simple from Tantoyuca, Veracruz, México. These bulbs were cultured in substrate (3 peat moss: 2 sand: 1 vermiculite) under a shade house at CIATEJ (Guadalajara, Jalisco, México) in the spring of 2013 and 2014.

### Controlled pollination

In order to find the various developmental stages of the megagametophyte ten non-pollinated flower buds of different sizes from 50 inflorescences of plants, which were randomly selected, collected and fixed. The rest of the buds remained attached to the inflorescence so that they continued their growth. At the time of anthesis the flowers were emasculated and covered with glassine paper to prevent uncontrolled pollination. Once the stigmas were receptive two non-pollinated flowers per inflorescence were selected and fixed. The remaining flowers were emasculated and hand pollinated with pollen from *P. tuberosa* var. Double and unripe fruits with different days after pollination (1 DAP-19 DAP) were collected in order to study the processes from fertilization to embryo development.

### Fixation

Ovules and immature seeds were extracted from the ovary and fixed in FAA (10:5:50:35 formaldehyde: acetic acid: ethanol: distilled water) for 24 h. After fixation, ovules were transferred to a 70 % ethanol solution and stored at 5 °C for later staining.

### Histological observation

Mayer’s hemalum methyl salicylate staining was used as a massive method for the analysis of large amounts of ovules and immature seeds, and the Feulgen staining method was used for confocal microscopy by using 3D projection series taken in “z”, only for those developmental stages where cells and tissues were positioned in different focal planes.

### Mayer’s hemalum-methyl salicylate stain-clearing (Stelly et al. 1984)

Specimens previously fixed and stored were stained with Mayer’s hematoxylin solution for 3 h at room temperature and later treated with heat for 30 min in a water bath at 40 °C, later, the specimens were treated with 2 % acetic acid for 40 min at 40 °C and then with 0.5 % acetic acid overnight in order to eliminate excess stain.

Thereafter, specimens were washed with 0.1 % sodium bicarbonate until the solution was clear, whereupon the solution was renewed and allowed to stand for 24 h. Finally, the specimens were subjected to an ethanol dehydration series: 25, 50, 70, 85, 95 % y 100 % for 15 min and 100 % ethanol for 2 h. The clarification of the tissue of was performed through a series of methyl salicylate:ethanol solutions of 3:1, 1:1, 1:3, for 1 h each (the specimens were stored in the last solution for 6 or more months at 5 °C).

For observation, the ovules were mounted on a solution of 100 % methyl salicylate and preparations analyzed under a Leica^®^ DMR microscope (Wetzlar, Germany) coupled to an EvolutionQEi^®^ camera (Media-Cybernetics, Bethesda, USA). The images were managed with the Image-Pro software (Media- Cybernetics, Bethesda, USA).

### Feulgen staining (Barrell and Grossniklaus 2005)

Ovules were treated with 1 M HCl for 1:30 h, then 5.8 M HCl for 2 h and again 1 M HCl for 1 h at room temperature. Thereafter, ovules were washed three times with distilled water and stained with Schiff solution for 3 h at room temperature and protected from light. Completed this standing time, the ovules were dehydrated in 30, 50, 70, 90 and 95 % ethanol for 30 min each and twice in 100 % ethanol. Finally, the ovules were allowed to stand overnight in a solution composed of 50 % ethanol and 50 % Leica immersion oil type F solution (Leica Cat. No. 11513859). Thereafter, ovules were mounted in 100 % Leica immersion oil type F for microscopic observation. Megagametophyte analysis was performed on a Leica TCS SPE RGBV confocal microscope, using a 532 nm laser excitation and a detection window between 555 and 700 nm. Images were captured and managed through the LAS X^®^ software (Leica Microsystems) with either 512 × 512 or 1024 × 1024 pixels. Images were processed with Adobe Photoshop version CS6, all Photoshop operations were applied to the entire image.

## Results and discussion

### Megasporogenesis

**Fig. 1:**
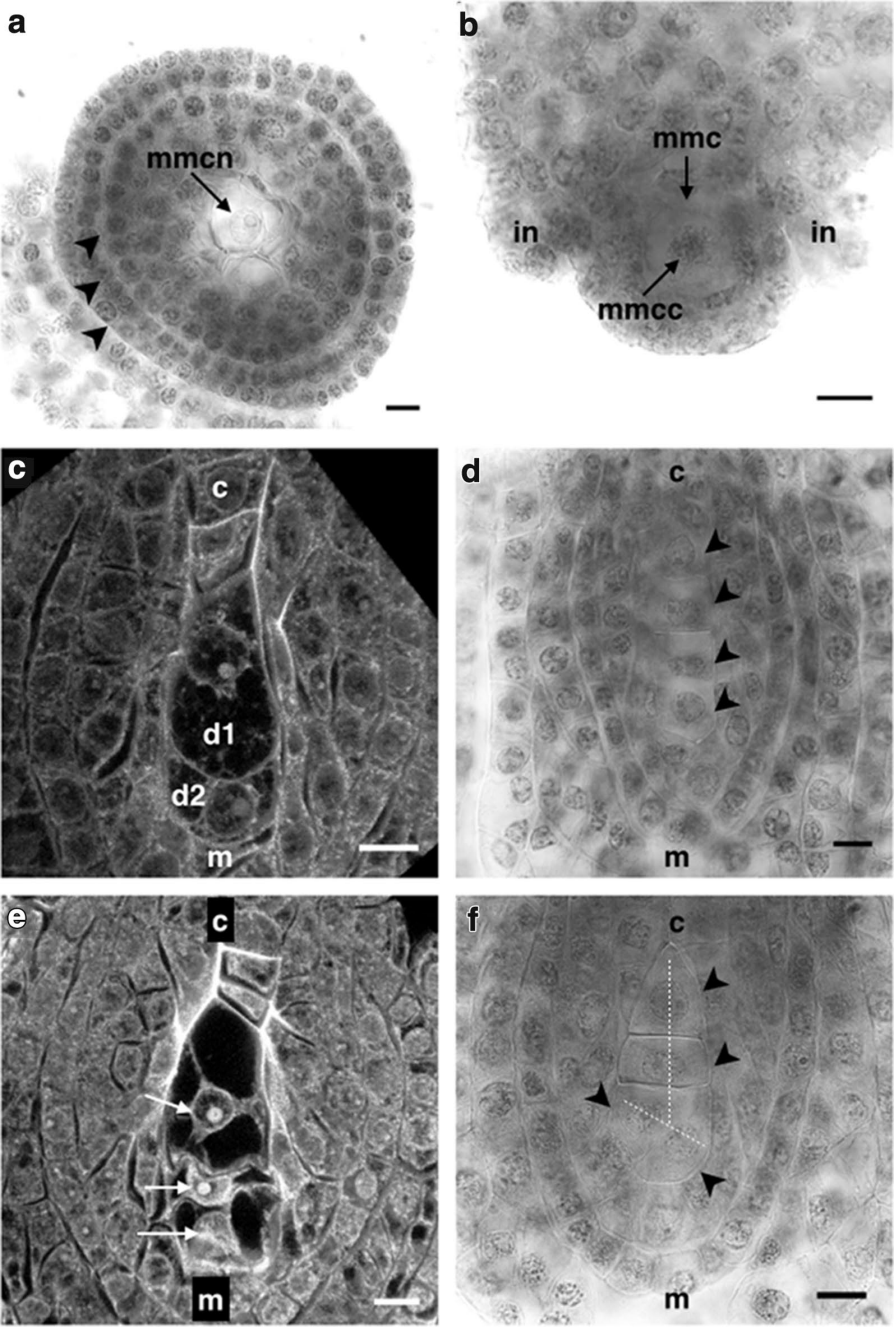
Megasporogenesis of *Polianthes tuberosa* var. Simple. **a** Cross-section of an ovule showing the condensed nucleus of a megaspore mother cell. **b** Megaspore mother cell located at the micropylar extreme of the ovule. **c** Diad. **d** Linear tetrad. **e** Triad. *Dark* or *black spaces* are vacuoles. **f** “T” shaped tetrad. *mmcn* megaspore mother cell nucleus, *mmcc* megaspore mother cell chromatin, *mmc* megaspore mother cell, *in* integuments, *arrow heads* in (**a**) sub-epidermic cells of the ovule, *c* chalaza, *m* micropyle, *d1* and *d2* diad cells, *white arrows* triad cells, *arrow head* in **d** and **f** cells of a tetrad, *dotted lines* in **f** “T” formation of a tetrad. *Bars* 10 µm

Megasporogenesis starts with the differentiation of an arquesporial cell that becomes the megaspore mother cell (MMC) which distinguishes from all other cells of the ovule primordia, since it has a larger size than the surrounding cells, its shape is circular to semicircular with an average diameter of 18.69 ± 2.33 μm, its nucleus is dense and well defined (Fig. 1a), and sometimes it is possible to observe the unorganized chromatin in the nucleus by way of filaments (Fig. 1b).

The MMC begins to increase in size and is seen shifted toward the micropylar end of the ovule. At this stage of development the integuments start to be differentiated (Fig. 1b). The diploid MMC is divided by meiosis generating in meiosis I a dyad of haploid cells of similar size or being the chalazal cell slightly larger than the micropylar cell (Fig. 1c), meiosis II results in a tetrad of cells commonly arranged in a linear manner parallel to the chalazal-micropylar axis (Fig. 1d). The average size of the tetrad is 52.95 ± 3.89 μm long and 19.07 ± 1.45 μm wide. Both integuments continued to grow surrounding the embryo sac and getting closer to the micropylar region.

Out of the total number of observations at this stage, 73.68 % of tetrads possessed a linear arrangement (Fig. 1d), however, the presence of other forms of arrangement was observed. In 21.05 % of tetrads of the remaining observations, the formation of tetrads in a “T” arrangement could be observed, where the two micropylar megaspores were found one beside the other or in an intermediate form in which the two megaspores closest to the micropyle are separated by an oblique division instead of a fully cross division (Fig. 1f).

The linear arrangement of the tetrad has been reported as a common pattern in several species of the order Aparagales as is the case of *A. fourcroydes*, *A. angustifolia* (Piven et al. 2001) and *A. tequilana* (Escobar-Guzmán et al. 2008; González-Gutiérrez et al. 2014). Nevertheless, some authors had reported the formation of linear tetrads and “T”, as in the case of *A. virginica* (Regen 1941), *A. lechuguilla* (Grove 1941) and *Y. aloifolia* (Wolf 1940). Watkins (1937) reported the frequent formation an intermediate configuration between linear and “T” arrangements for *Yucca* rupicola similar to that reported in the present study for *P. tuberosa* var. Simple.

Moreover, in some isolated observations (5.26 %) the formation of triads instead of tetrads was observed (Fig. 1e), as those reported by Regen (1941) in *A. virginica* who interpreted the presence of triads as the possible non division of one of the megaspores of the dyad to enter meiosis II. Gomez-Rodríguez et al. (2012) observed the formation of triads in the pollen microsporogenesis of *A. tequilana* and *A. angustifolia* which are formed by a mechanism where a failure in meiosis II in a cell of the dyad prevented the formation of cell wall in the daughter nuclei, which finally are restored, leading to the formation of a 2n microspore and two n microspores (unreduced gametes).

### Megagametogenesis

**Fig. 2:**
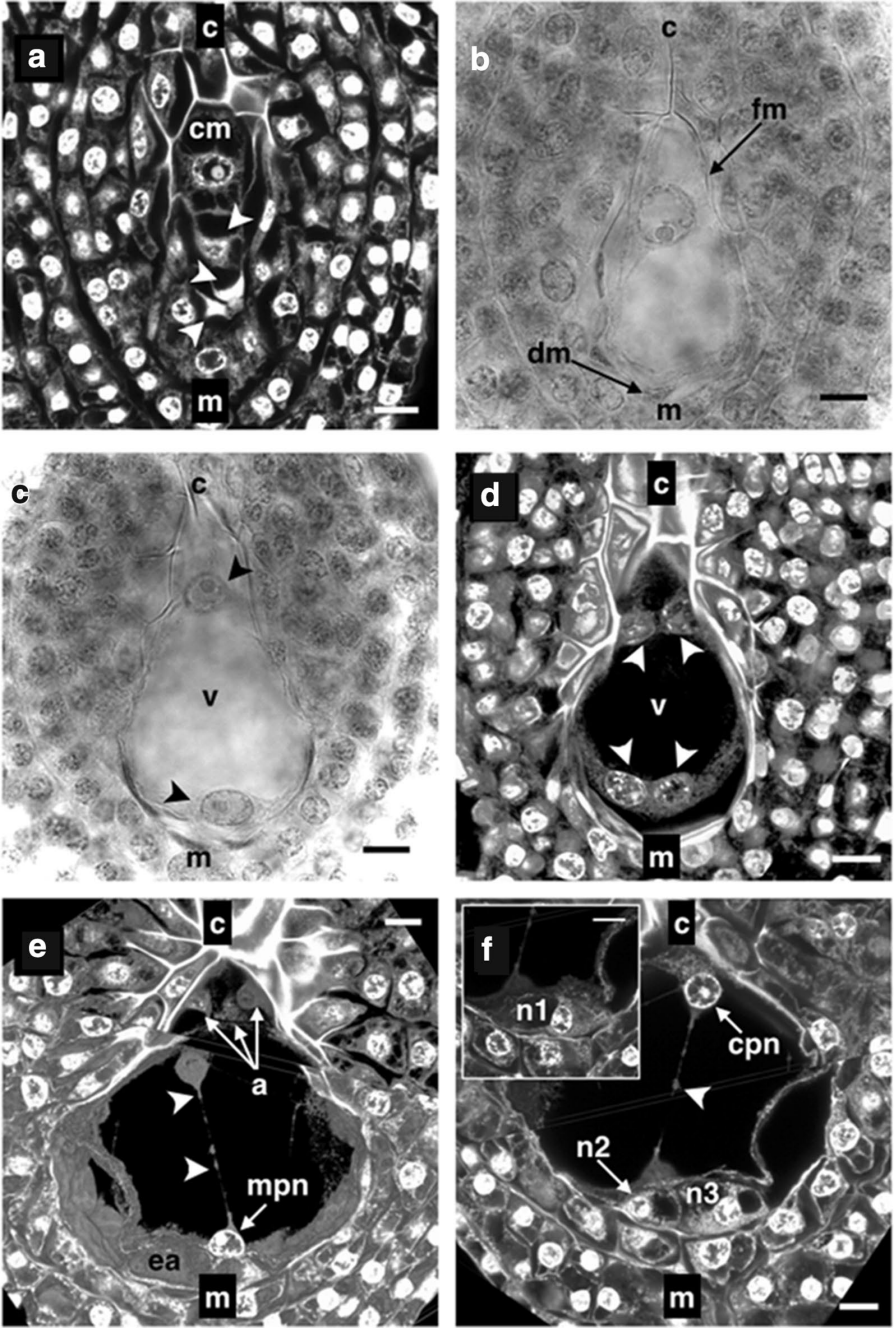
Megagametogenesis of *Polianthes tuberosa* var. Simple. **a** Linear tetrad. The three closest cells to the micropylar extreme are degenerating. **b** Functional megaspore. **c** First mitotic division. **d** Embryo sac with four nuclei. **e** and **f** Third mitotic division of the embryo sac with eight nuclei. *cm* chalazal megaspore, *fm* functional megaspore, *dm* degenerating megaspores, *v* vacuole, *a* antipodal cells, *ea* egg apparatus, *mpn* micropylar polar nucleus, *cpn* chalazal polar nucleus, *n1*, *n2* and *n3* cells that will form the egg apparatus, *c* chalaza, *m* micropyle, *arrow heads* in (**a**) megaspores being degraded, *arrow heads* in (**c**) primary chalazal and micropylar nuclei, *arrow head* in (**d**) nuclei produced by the second meiotic division of the embryo sac, *arrow heads* in (**e**) and (**f**) cytoplasmic filaments. *Bars* 10 µm

In the normal development of the tetrad, three of the megaspores, the closest to the micropylar end degenerated while the chalazal cell remained intact (Fig. 2a), becoming the functional megaspore (FM) (monosporic pattern), similar to what was found in *Y. aloifolia* (Wolf 1940), *A. virginica* (Regen 1941) and *A. tequilana* (Escobar-Guzmán et al. 2008; González-Gutiérrez et al. 2014); whereas in the species *Y. filamentosa* Reed (1903) reported that is the second megaspore in the chalazal-micropylar direction that remains while the other three megaspores degrade. Meanwhile, Piven et al. (2001) mentioned that in the species *A. fourcroydes* and *A. angustifolia* the development of the embryo sac is given from two of the megaspores closest to the chalazal end; this developmental pattern is called Bisporic Allium type, similar to that present in other representative species of the Aparagaceae family as *Scilla persica* (Svoma and Greilhuber 1987).

The FM possesses a large and well-defined nucleus which is usually located at the first or second third of the developing embryo sac (chalazal-micropylar direction) (Fig. 2b). The FM underwent a first mitotic division forming a binucleate sac (62.42 ± 6.90 μm long and 39.11 ± 5.40 μm wide), the newly formed nuclei migrated one towards the chalazal end and the other to the micropylar end of the sac, both being separated by a large central vacuole (Fig. 2c). Once at the ends, a second mitotic division generated a sac with four nuclei (two at each extreme), which were located very close to the walls of the embryo sac and continued separated by the central vacuole which like the sac shows a continuous increase in size measuring 77.31 ± 5.96 μm long and 54.62 ± 5.98 μm wide (Fig. 2d).

The mitotic division of the nuclei occurred synchronously at both poles of the sac in the same way that occurs in *A. tequilana* (González-Gutiérrez et al. 2014) and contrary to what was reported by Grove (1941) for *A. lechuguilla* where mitotic division first occurs in the nuclei located at the micropylar end of the embryo sac. Moreover, at this stage the formation of the hypostase became evident, a tissue that is formed immediately above the FM which is apparently formed by a group of cells with thickened cell walls that are easily observed under the optical and confocal microscope because they are stained more intensely than the rest of cells, such cell formation seems to be connected to the vascular bundles of the ovule through the nucellar tissue.

According to Tilton (1980) the formation of the hypostase occurs during the meiotic-mitotic interface of the FM. In this regard, Tilton proposed that the main function of such structure focuses on nutrient translocation into the embryo sac before and after fertilization. Hypostase formation is reported as a frequent character in Agavaceae (now sub-family Agavoideae) (Tilton and Mogensen 1980) and other families in the group of monocots (Rudall 1997).

A third mitotic division resulted in an embryo sac with eight nuclei, four at each end of the sac and as in the second mitotic division it happened synchronously. At this time the chalazal haustorial tube became more apparent at the end of the sac and is where three of the four newly formed nuclei were located. The remaining nucleus was placed immediately beneath them, while at the opposite end, all four micropylar nuclei were aligned to the embryo sac wall (Fig. 2e, f).

Finally, one of the four nuclei located at the micropylar end became the micropylar polar nucleus and started to migrate through the central vacuole toward the chalazal end of the sac to meet the single nucleus which is observed outside the haustorial tube, said nucleus became the chalazal polar nucleus. When a 3D reconstruction was performed thin filaments were observed connecting the two polar nuclei (Fig. 2e, f). According to Tilton and Lersten (1981) these filaments are formed of cytoplasm and provide the vehicle by which the polar nuclei can join. Ikeda (1902) supported the hypothesis that these cytoplasmic connections found between different cell types in the embryo sac provide the means by which the antipodals, the central cell and the egg apparatus remain in communication.

Up to this stage of development, the embryo sac exhibited an ovoid to pyriform shape being narrower at the chalazal end and wider toward its micropylar end (Fig. 2e, f). In the chalazal extreme the development of a narrow tube called haustorial tube was observed (Fig. 4b), which is similar to that reported by Tilton (1978) in *Ornithogalum caudatum* where the hypostase is surrounding the haustorial tube. The haustorial tube was observed as an invagination into the nucellar tissue of the ovule so several authors attribute functions of nutrition to the embryo sac (Reed 1903; Watkins 1937; Wolf 1940; Rudall 1997).

### Characterization of the mature embryo sac

**Fig. 3:**
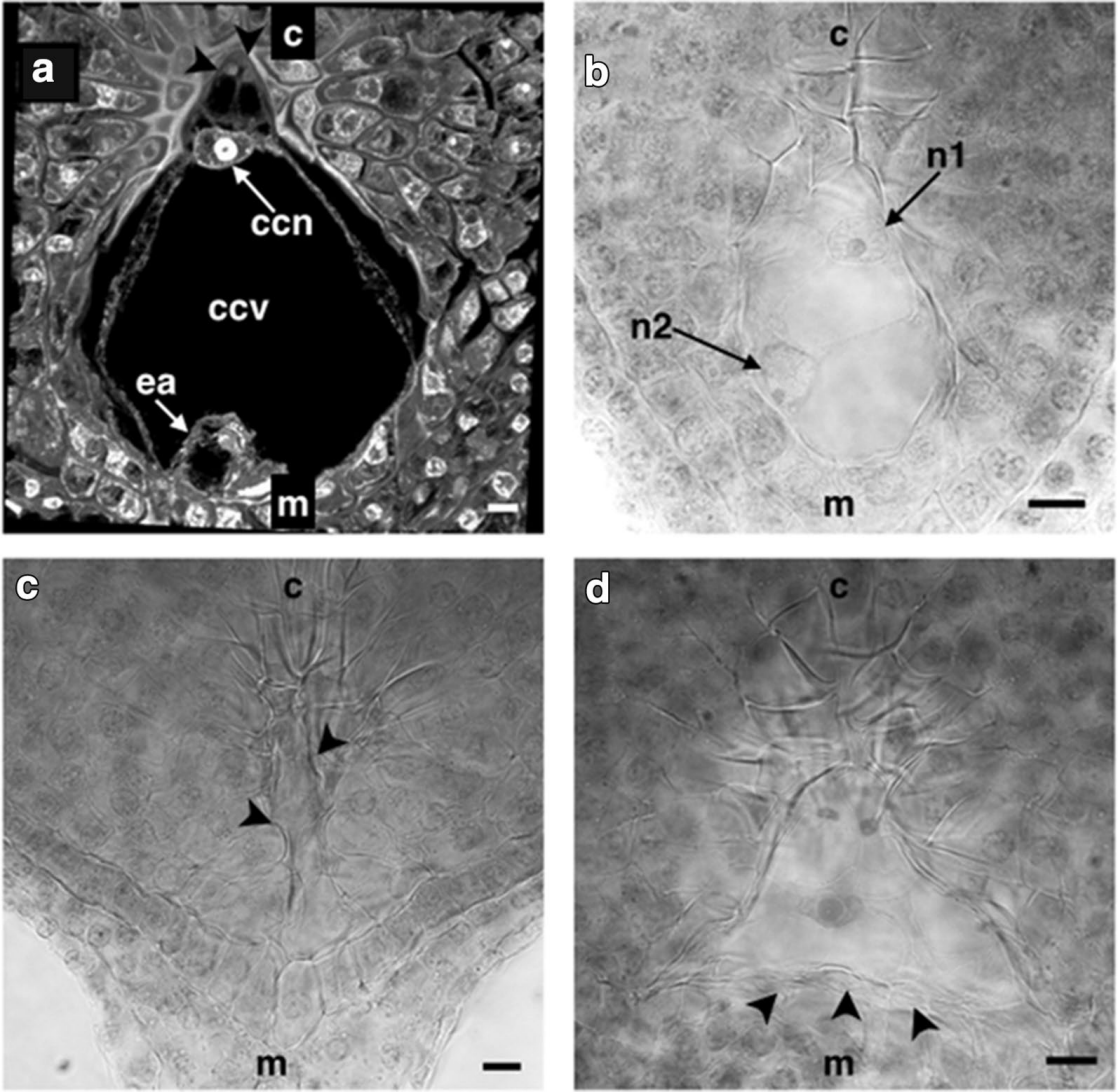
Mature embryo sac of *Polianthes tuberosa* var. Simple. **a** Polygonum type normal embryo sac development. **b**–**d** Abnormalities found in mature embryo sacs. **b** Embryo sac with retarded development with only two nuclei. **c** Colapsed embryo sac. **d** Embryo sac without egg apparatus and abnormal growth of the uniseriate nucellar tissue. *ccn* central cell nucleus, *ccv* central cell vacuole, *ea* egg apparatus, *n1* and *n2* nuclei, *ch* chalaza, *m* micropyle, *arrow heads* in (**a**) antipodal cells, *arrow heads* in (**c**) collapsed embryo sac, *arrow heads* in (**d**) abnormal growth of uniseriate nucellar tissue. *Bars* 10 µm

With the migration of one of the micropylar nuclei toward the chalazal, the embryo sac soon acquired its final shape and its nuclei became cellularized; being the three cells in the haustorial tube the antipodals, the two nuclei located below the haustorial tube became the polar nuclei contained in the central cell and the three cells located at the micropylar end became the egg apparatus so that the normal development of the embryo sac of *P. tuberousa* var. Simple was typified as monosporic Polygonum-type (Fig. 3a) as described by Maheswari (1937, 1948).

Out of the total samples analyzed at this stage of development 81.66 % corresponded to this pattern, and the average size of the mature sac was 152.02 ± 5.54 μm long by 129.74 ± 5.41 μm wide; in the rest of the samples the presence of defects and/or abnormalities in the development of the embryo sac was detected. These abnormalities were classified into three main groups:

- Embryo sacs in retarded stages of development, i.e. embryo sacs that did not corresponded to the stage of development found in the remaining ovules from a single ovary (Fig. 3b) and corresponded to 1.66 % of analyzed samples.
- Collapsed embryo sacs where degradation of the embryo sac and the nucellar tissue was observed in 10 % of the samples (Fig. 3c).
- Embryo sacs where the formation of the egg apparatus is not observed due to an abnormal thickening of the nucellar cell layer lining the ovule in its micropylar end. These embryo sacs usually lost their pyriform shape showing a “boomerang” shape (Fig. 3d). This malformation group corresponded to 6.66 % of the specimens analyzed.

Regen (1941) described the presence of a large amount of “unproductive” ovules in *A. virginica* where reproductive cells were not formed due to degeneration of nucellar and sporogenesis tissues. Meanwhile, Cappelletti (1927) reported the presence of a brief hypertrophy of the nucellar nuclei followed by cell degeneration of this tissue ending with the collapse of the embryo sac.

### Antipodal cells

**Fig. 4:**
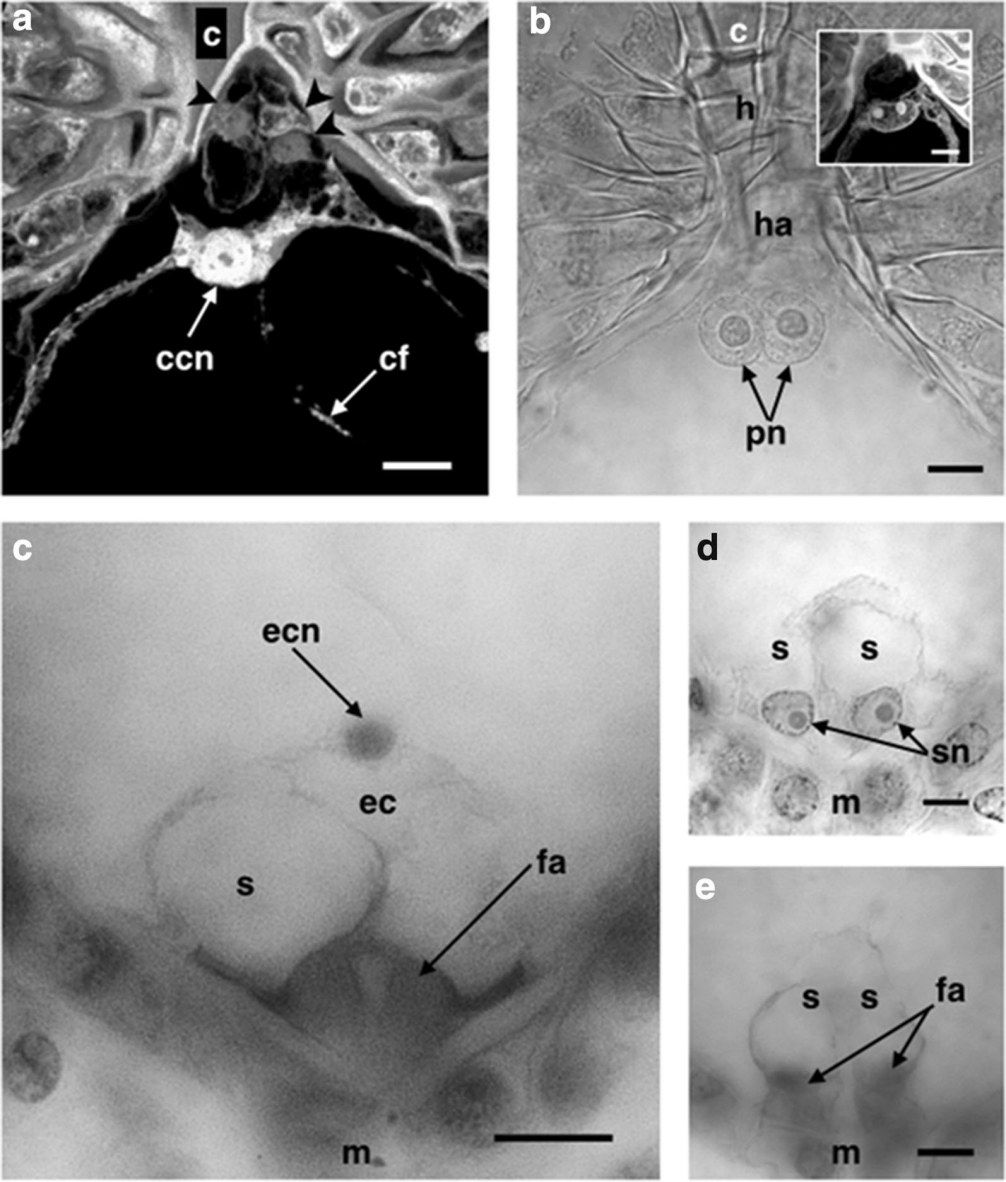
Cell types of the mature embryo sac of *Polianthes tuberosa* var. Simple. **a** Antipodal cells in the haustorial tube. **b** Polar nuclei before karyogamy. **c** Egg apparatus and the filiform apparatus in the background. **d** Synergid cells. **e** Filiform apparatus. *ccn* central cell nucleus, *cf* cytoplasmic filaments, *h* hypostase, *ha* haustorial tube, *pn* polar nuclei, *ec* egg cell, *ecn* egg cell nucleus, *s* synergid cells, *sn* synergid nuclei, *fa* filiform appa-ratus, *c* chalaza, *m* micropyle, *arrow heads* in (**a**) antipodal cells. *Bars* 10 µm

The antipodals were observed as three cells smaller than the rest of the mature embryo sac cells (7.68 ± 0.34 μm long by 6.46 ± 0.43 μm wide). These were located inside the haustorial tube and showed a triangular morphology usually with their nuclei polarized towards the chalazal end of the sac (Figs. 3a, 4a).

Sometimes it was not possible to detect the presence of the antipodal cells so their behavior inside the sac appears to be variable being disintegrated before karyogamy of the polar nuclei (Fig. 4b) or remain intact even after the moment of double fertilization. According to Tilton (1978), the antipodals are unique cells that vary in their behavior within the mature female gametophyte, the only trait they share with each other is their location in the chalazal end of the sac; these cells can be ephemeral and disintegrated shortly after its formation as in the case of *A. virginica* (Regen 1941).

In *Tofieldia glutinosa*, the antipodals even proliferate in the maturation stage of the embryo sac, being up to eight antipodal nuclei (Holloway and Friedman 2008); another example is seen in most of the members of the Poaceae family where the number of antipodals varies between six and 300 (Anton and Cocucci 1984). Misinterpretations in studies of the female gametophyte of some species have been due to the difficulty in visualizing the antipodals by examination under an optical microscope, mainly due to its chalazal position in the embryo sac, especially when they are contained within structures such as haustoria (Maheswari 1948, 1950). Recently, Song et al. (2014) confirmed the persistence of the three antipodals after double fertilization in *Arabidopsis* by expression of fluorescence reporter genes.

### Central cell (fusion of polar nuclei)

The polar nuclei were very similar to each other, they had a spherical to semispherical shape and a size of approximately 10.17 ± 1.48 μm diameter. According to Tilton (1980), both nuclei have a similar size and morphology such that it is difficult to distinguish from each other (Fig. 4b), however, Maheshwari (1941) considered that the original nucleus of the micropylar end may become larger than the polar nucleus from the chalazal end.

The distance between the polar nuclei decreased until they were beside each other, and their membranes entered in contact getting fused, and sometimes a single nucleus with two nucleoli inside could be observed (Fig. 4b). Finally, as a result of the polar nuclei karyogamy the nucleus of the central cell was generated (Fig. 4a). The nucleus of the central cell was of a semicircular or ovoid shape with an average size of 15.85 ± 1.11 μm long by 16.41 ± 1.21 μm wide.

The nucleus of the central cell as well as the polar nuclei retained its polarity to the chalazal end of the sac (Fig. 4a). In the Agavaceae, this polarity was similarly observed in *A. fourcroydes* and *A. angustifolia* (Piven et al. 2001), *A. lechuguilla* (Grove 1941) *A. tequilana* (González-Gutiérrez et al. 2014) and *Y. rupicola* (Watkins 1937). However, according to Tilton (1978, 1980) the polar nuclei of the central cell of most angiosperms migrate toward the center of the sac, as in the case of maize (Huang and Sheridan 1994) and *Arabidopsis thaliana* (Olsen 2004). According to Maheswari (1950), the position of the nucleus of the central cell towards the chalazal end of the embryo sac is an indication that an helobial type of endosperm will be developed once double fertilization took place.

### Egg apparatus

The egg apparatus is located at the micropylar end of the embryo sac and is composed of three cells, two synergids and the egg cell (Fig. 4c). The synergids have a very similar shape between them, their nuclei are highly polarized towards the micropyle and a large vacuole is observed towards the chalazal end (Fig. 4d). Their average size is 16.76 ± 0.30 μm long and 12.92 ± 0.47 μm wide. One of their walls is in contact with the edge of the embryo sac, however, they are separated from each other by a small space (Fig. 4e) and it was possible to observe the filiform apparatus at the base of both synergids (Fig. 4e).

The egg cell was highly polarized with a dense nucleus at the chalazal end and the vacuole at the micropylar end (Fig. 4c). This polarity is found in a rather frequent manner in most angiosperms as in the case of *Nicotiana tabacum* (Mogensen and Suthar 1979; Tian et al. 2005), however, some times the nucleus could be located at the second third of the egg cell with a large number of small vacuoles distributed around (Russell 1993). The dimensions of the egg cell were in an average of 25.96 ± 1.60 μm long and 22.89 ± 1.59 μm wide.

### Double fertilization

**Fig. 5:**
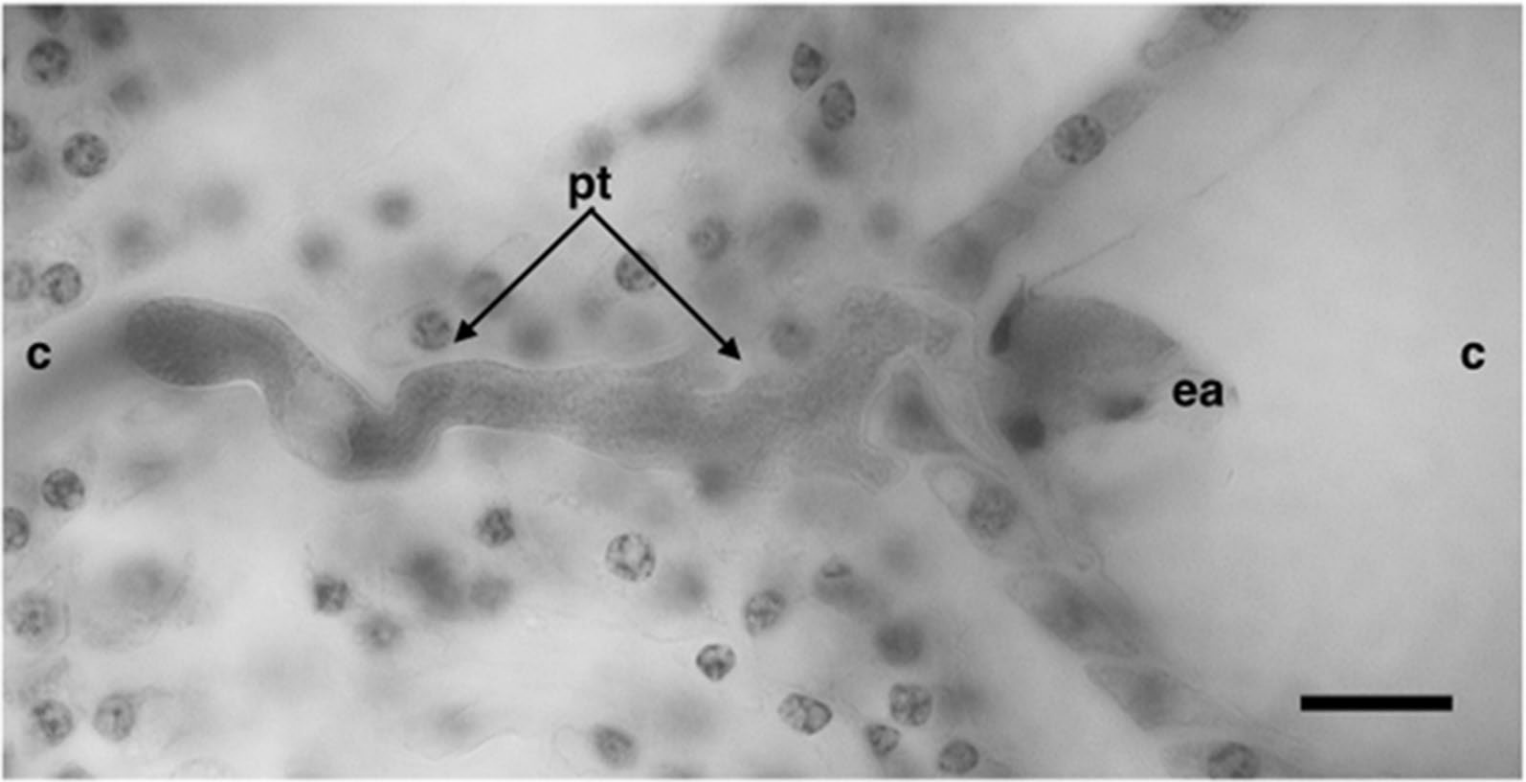
The beginning of fertilization of *Polianthes tuberosa* var. Simple. A pollen tube entering the embryo sac through the micropyle. *pt* pollen tube, *ea* egg apparatus, *c* chalaza, *m* micropyle. *Bars* 20 µm

The initiation of the process of double fertilization was observed in ovules collected on 6 DAP. The pollen tube that remained attached to the integuments could be observed at the micropyle. The pollen tube made its way through the outer and inner integuments and then reached the micropylar end of the embryo sac through the cells of the uniseriate nucellar tissue to make contact with the cells of the egg apparatus within embryo sac (Fig. 5). Further studies on the double fertilization process will be published elsewhere.

### Zygote formation and embryo development

**Fig. 6:**
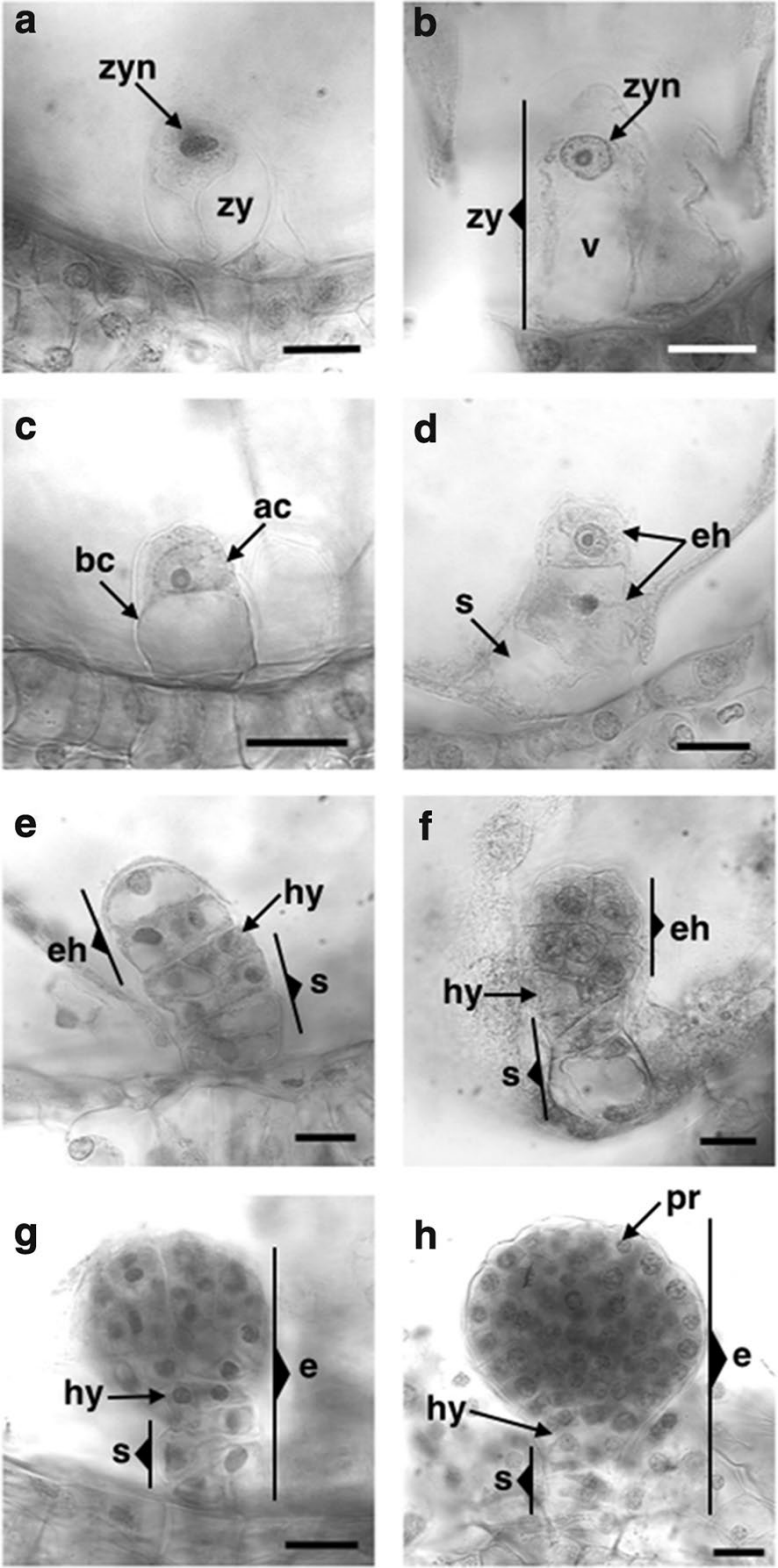
Early embryo development in *Polianthes tuberosa* var. Simple. **a** Zygote. **b** Zygote elongation. **c** First cell division of the zygote. **d** First cell division of the embryo head. **e** Second cell division of the embryo head. **f** Eight cell embryo. **g** Early globular embryo. **h** Globular embryo. *zy* zygote, *zyn* zygote nucleus, *v* vacuole, *ac* apical cell, *bc* basal cell, *eh* embryo head, *s* suspensor, *hy* hypophysis, *pr* protoderm, *e* embryo. *Bars* 20 µm

As a result of fertilization of the egg cell by one of the sperm nuclei at 7DAP it was possible to observe the formation of the zygote (Fig. 6a). The zygote showed a semi-spherical shape and an increase in size is observed relative to the size shown by the egg cell prior to fertilization. Zygote dimensions are 44.33 ± 1.28 μm long by 38.48 ± 1.33 μm wide. The zygote nucleus is relocated to its position at the chalazal end of the cell as it was in the egg cell, however, prior to its final position, the nucleus of the egg cell moves toward the center of the cell, putatively at the moment of its fertilization (to be published elsewhere). This polarity shown by the zygote of *P. tuberosa* is similar to the studies on zygotic embryogenesis performed in model plants such as *Capsella bursa*-*pastoris* (Schulz and Jensen 1968), *Nicotiana tabacum* (Mogensen and Suthar 1979) and *A. thaliana* (Mansfield and Briarty 1991; Mansfield et al. 1991).

At 7 DAP it was also possible to observe how the zygote began to elongate and changed from hemispherical to oval shape, so that its dimensions throughout the longitudinal axis increased to 55.80 ± 1.15 μm (Fig. 6b) while the width of the zygote remained constant (38.85 ± 0.82 μm). The polarity of its nucleus was kept oriented to the chalazal end of the cell. This elongation of the zygote is seen as a common feature in angiosperms, which prepares the embryo for the first cell division. In the case of *A. thaliana* (Mansfield and Briarty 1991) the zygote showed an elongation of approximately three times its size (in apical-basal direction) before the first division, and in the case of *A. tequilana* this increase is a third the original size of the zygote (González-Gutiérrez et al. 2014).

Once the cell forming the zygote is elongated, this is divided transversely at the chalazal-micropylar axis resulting in two cells, the apical cell and the basal cell (8DAP) (Fig. 6c). The division of the zygote in a transversal direction observed in *P. tuberosa* var. Simple occurred similar to that observed in the vast majority of angiosperms (Rodríguez-Garay et al. 2000; Lau et al. 2012), however, this division can be given longitudinally (Johri and Rao 1984) or oblique as in the case of wheat (Batygina 1978).

The first division of the zygote generated an apical cell, which showed a large and highly condensed nucleus and a basal cell with a large vacuole that covers virtually the entire space of the cell (Fig. 6c). This first division occurred asymmetrically so the apical cell was usually smaller (19.81 ± 0.65 μm long by 23.79 ± 1.80 μm wide) than the basal cell (38.94 ± 2.11 μm long by 29.83 ± 2.85 μm wide) as in *A. tequilana* (González-Gutiérrez et al. 2014) and *A. thaliana* (Mansfield and Briarty 1991) where the cell plate development generates a smaller apical cell compared to the basal cell.

In the analyzed samples at 8 DAP the first division of the apical cell that forms the embryonic head was observed (Fig. 6d), this division as it was for the first division of the zygote occurred transversely, contrary to what was reported for *A. thaliana* where this division occurs longitudinally (Mansfield and Briarty 1991; Capron et al. 2009). The apical cell continued to divide, generating a four-celled embryo by a longitudinal division. On the other hand, the basal cell through a series of transversal divisions following the chalazal-micropylar axis formed the embryonal suspensor, which in turn at this stage of development was able to form the hypophysis from the first division of the basal cell (9 and 10 DAP) (Fig. 6e). By the 10th and 11th DAP the studied samples showed the formation of eight-celled embryos (Fig. 6f), similar to those described by Batygina (1978), where not only the first division of the zygote, but all subsequent divisions of the embryo occurred obliquely to the chalazal-micropylar axis. The divisions of the embryo continue until the embryo reached the early globular stage (12 and 13 DAP) with approximately 16 cells (Fig. 6g), and finally the formation of globular embryos probably 64 cells or more, stage from which the protoderm differentiation can be observed (16 DAP) (Fig. 6h).

### Endosperm development

**Fig. 7:**
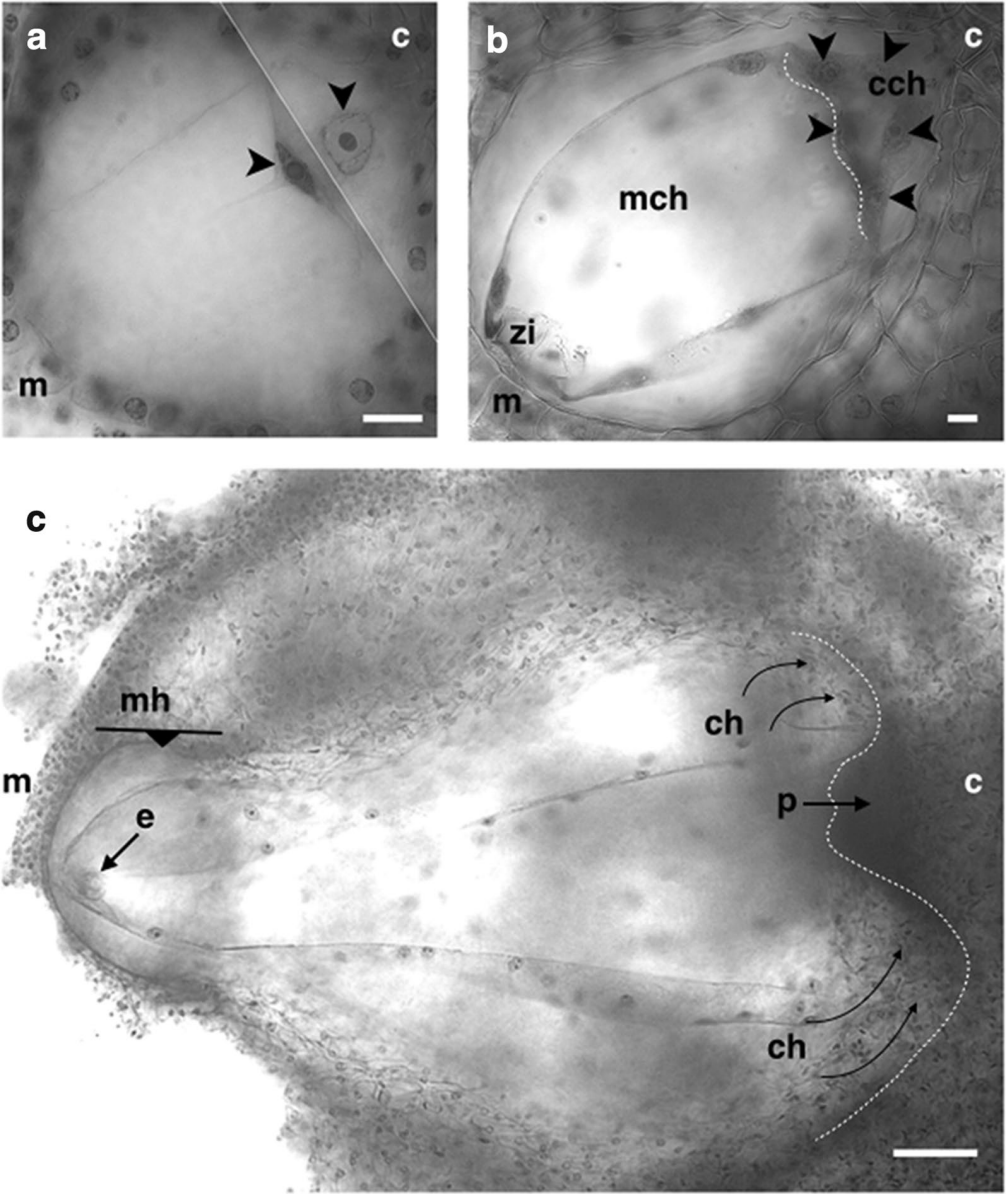
Helobial endosperm development of *Polianthes tuberosa* var. Simple. **a** First division of the endosperm mother cell. **b** Development of the chalazal and micropylar chambers. **c** Development of two chalazal haustoria divided by the postament. *zy* zygote, *e* embryo, *cch* chalazal chamber, *mch* micropylar chamber, *p* postament, *ch* chalazal haustorium, *mh* micropylar haustorium, *c* chalaza, *m* micropyle, *arrow heads* in a result of the first division of the endosperm mother cell, *arrow heads* in **b** endosperm cell and nuclear divisions, *dotted line* in **b** formation of the cell wall dividing the micropylar and chalazal chambers, *dotted line* in **c** formation of two chalazal haustoria divided by the postament, *curved arrows* haustoria pushing towards the chalaza; *The line (**a**) indicates the superposition of two images taken at different focus levels of the same specimen. *Bars* in **a** and **b** 20 µm. *Bar* in **c** 100 µm

Along with the changes presented in the zygote, as a consequence of fertilization of the central cell by the second sperm nucleus, the endosperm mother cell was generated, which made a first division transverse to the axis chalazal-micropylar forming two cells, one cell confined to the area of the chalazal haustorium and the second cell was located throughout the embryo sac in the last two thirds thereof (Fig. 7a). The chalazal cell then followed a series of divisions first of the nuclear type and then of the cellular type forming a small chalazal chamber, meanwhile the micropylar nucleus generates a second micropylar chamber which is of a larger size and where endosperm divisions occur in a nuclear way where most of endosperm nuclei are located at the periphery of the embryo sac (7 DAP) (Fig. 7b).

The formation of both chambers, the endosperm development of the nuclear type in the micropylar chamber (Bhojwani and Bhatnagar 1983; Floyd and Friedman 2000), as well as the aforementioned chalazal position of the central cell nucleus in the embryo sac are typical features of the helobial type of endosperm (Maheshwari 1950), thus *P. tuberosa* var. Simple developed an endosperm that was classified as of helobial type, like those reported in the species *Hesperocallis undulata* (Cave 1948) and *A. tequilana* (González-Gutiérrez et al. 2014).

Endosperm development was observed at an early zygote stage so it was possible to observe several divisions of the same before the first division of the zygote took place (Fig. 7b). The endosperm development in *P. tuberosa* was similar to that of *Amaranthus hypocondriacus* (Coimbra and Salerma 1999), *T. glutinosa* (Holloway and Friedman 2008) and *A. tequilana* (González-Gutiérrez et al. 2014).

The general shape of the embryo sac started to change, the walls of the sac moved toward the nucellar tissue and lost its pyriform appearance, and taking an ovoid shape slightly narrow at the chalazal end where the chalazal haustorial tube was originally placed (Fig. 7b). At 8 DAP important changes began to be observed. The embryo sac continuously changed its shape as a result of increase in volume and divisions of the cells of the endosperm, the chalazal walls of the embryo sac pushed the chalazal nucellar tissue generating two new haustoria which were divided by the postament, a tissue containing a set of thickened cells that formed the hypostase in the unfertilized ovule. Then a third haustorium was formed in the micropylar area of the embryo sac where the embryo develops (Fig. 7c). The development of haustoria both chalazal and micropylar after fertilization are characteristics that are commonly observed in several species of the order Asparagales (Rudall 1997). As stated before, the differentiation of the embryo protoderm could be observed at 16 DAP, however, even though endosperm cellularization was not observed, it might occur after this stage.

## Conclusions

The normal development of the *P. tuberosa* var. Simple embryo sac follows a monosporic pattern of the Polygonum type and starts its development from the chalazal megaspore. At maturity, the embryo sac is of a pyriform shape with a chalazal haustorial tube where the antipodals are located, just below the hypostase, which connects the embryo sac with the nucellar tissue of the ovule. The central cell nucleus shows a high polarity, being located at the chalazal extreme of the embryo sac. Due to this particular characteristic, the second sperm nucleus has to travel a long distance in order to fertilize such nucleus. The position of cells inside the *P. tuberosa* embryo sac may be useful for in depth studies about the double fertilization. Furthermore, it was possible to make a chronological description of the events that happen from fertilization and early embryo development to the initial development of the endosperm which was classified as of the Helobial type.
